# Complete Genes May Pass from Food to Human Blood

**DOI:** 10.1371/journal.pone.0069805

**Published:** 2013-07-30

**Authors:** Sándor Spisák, Norbert Solymosi, Péter Ittzés, András Bodor, Dániel Kondor, Gábor Vattay, Barbara K. Barták, Ferenc Sipos, Orsolya Galamb, Zsolt Tulassay, Zoltán Szállási, Simon Rasmussen, Thomas Sicheritz-Ponten, Søren Brunak, Béla Molnár, István Csabai

**Affiliations:** 1 Molecular Medicine Research Group, Hungarian Academy of Sciences, Budapest, Hungary; 2 Children's Hospital, Harvard Medical School, Boston, Massachusetts, United States of America; 3 Department of Physics of Complex Systems, Eötvös University, Budapest, Hungary; 4 Department of Animal Hygiene, Herd Health and Veterinary Ethology, Szent István University, Budapest, Hungary; 5 2nd Department of Internal Medicine, Semmelweis University, Budapest, Hungary; 6 Center for Biological Sequence Analysis, Technical University of Denmark, Lyngby, Denmark; 7 Department of Physics and Astronomy, The Johns Hopkins University, Baltimore, Maryland, United States of America; Yale School of Public Health, United States of America

## Abstract

Our bloodstream is considered to be an environment well separated from the outside world and the digestive tract. According to the standard paradigm large macromolecules consumed with food cannot pass directly to the circulatory system. During digestion proteins and DNA are thought to be degraded into small constituents, amino acids and nucleic acids, respectively, and then absorbed by a complex active process and distributed to various parts of the body through the circulation system. Here, based on the analysis of over 1000 human samples from four independent studies, we report evidence that meal-derived DNA fragments which are large enough to carry complete genes can avoid degradation and through an unknown mechanism enter the human circulation system. In one of the blood samples the relative concentration of plant DNA is higher than the human DNA. The plant DNA concentration shows a surprisingly precise log-normal distribution in the plasma samples while non-plasma (cord blood) control sample was found to be free of plant DNA.

## Introduction

We are constantly exposed to foreign DNA from various sources like benign or malicious microbes in and on our body, pollens in the inhaled air and as the largest amount with the daily food supply. DNA molecules are ubiquitous in large numbers in all raw and unprocessed food. Depending on the extent of processing, various fractions of DNA molecules of varying size may be present in the consumed product, even in processed food such as corn chips and chocolate [1].

Uptake and fate of foreign DNA ingested with the daily food intake in the gastrointestinal tract of mammals is not a completely understood topic. Though exogenous nucleotides are essential at least for maintaining host immunity to allergenic tissues and restoring specific immune responses to foreign antigens [2], the amount of DNA in food is relatively low compared to other constituents and does not have significant nutritional value, hence nutritional studies rarely deal with this issue. The final step of uptake of nucleotides in the epithelium of the gastrointestinal tract is a relatively well understood complex process [3]. In contrast, the comprehension of the degradation process of long chains of DNA and possible uptake of larger fragments face many methodological challenges and very few studies have been conducted on the digestion of food-derived DNA within the 68 m long digestive tract of adult humans [1]. Animal feeding studies have demonstrated that a minor amount of fragmented dietary DNA may resist the digestive process (for a recent review see [4]) and there are sporadic reports in the literature claiming that orally administered small fragments of bacterial DNA [5] or plant RNA [6] can transgress the intestinal barrier, but no studies have explored the question if large DNA segments can pass from natural food intake to the circulatory system.

Blood is not free of DNA. White blood cells have nuclei that contain genetic material, which gives the dominant part of the DNA in a full blood sample. Beyond the DNA contained in the white blood cells the cell free blood plasma contains DNA, too. This is the so called circulating cell-free DNA (cfDNA) which is an ideal target to test the presence of foreign DNA, since most of the human “background” is removed by the cellular fraction.

### Characteristics of Cell-free DNA

Circulating cell-free DNA (cfDNA) is defined as extracellular DNA occurring in body fluids was discovered in the human bloodstream and first described in 1948 by Mandel and Metais [7], but its origin and possible role is still controversial. The cfDNAs are mostly double-stranded molecules with fragment size in a wide range from 180 bp up to 21 kbp [8], [9]. The shorter fragments are thought to be related to the histone octamer structure and apoptotic degradation process, while necrosis results much larger fragments. Through phagocytosis of apoptotic cells macrophages may release the degraded DNA fragments into the bloodstream. These cfDNA fragments circulate as nucleoprotein complexes and in healthy individuals, the main part of cfDNA is found adsorbed to the surface of blood cells [10], [11].

The cfDNA concentration in healthy people is between 0 and 100 ng/ml with a mean of 13
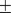
3 ng/ml. This level is increased by an order of magnitude in various types of cancer up to a mean of 180
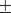
38 ng/ml [12]. How the circulating cfDNA is then eliminated from the blood remains unknown in general but altered nucleotide metabolism was observed in tumorous patients. According to this hypothesis the increased cfDNA concentration is caused by the reduced DNase activity in the tumorous plasma [13] and indeed treatment of tumorous mice with ultra low doses of nucleases significantly decreased the liver and lung metastasis [14]. On the other hand according to Holdenrieder et al. [15] the efficiency of plasma nucleases is limited because the structure of nucleoprotein complexes is able to protect the cfDNA from degradation.

Studying the clearance of fetal DNA from maternal blood after birth by Lo et al. [16] a relatively quick mean half-life time (16.3 min, range 4–30 min) of the cfDNA was observed by using PCR. During the elimination process an initial rapid tissue uptake phase and a second DNase-mediated slower phase can be separated [16], [17].

The cfDNA fragments circulating in the plasma are a mostly uniform sample of the whole genome, however there are some over-represented fragments. Increased DNA integrity was observed in tumorous plasma samples due to the higher ratio of 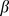-actin fragments with lengths of 400 bp compared to samples from patients with non-neoplastic diseases, which may be caused by the different origin and degradation rate of the cfDNA [18].

### The Origin of the Cell Free DNA

There are many, sometimes contradicting, theories concerning the release of cfDNA and its distribution in the body. Also, we are only at the first steps to uncover the cellular and molecular mechanisms that transfers cfDNA from cells to blood. Initially pathogen origin has been attributed to cfDNA, later different pathological conditions like cancer, inflammation and autoimmune disease, while finally it has been shown to be present in the plasma of subjects with normal physiological conditions [19], [20], too. Our current understanding is that apoptotic cells – which are present in healthy individuals, too – are the primary source. Additionally, in different diseases (inflammation, autoimmune, trauma and cancer) necrotic cells may increase the cfDNA level [8], [21].

There is an alternative theory, which suggests that white blood cells are the main source of cfDNA. Lee et al. [22] attributes the higher concentration in serum than plasma samples to the process of clotting caused by the lysis of white blood cells. Also, in limphocyte, DNA with lower molecular weight than genomic DNA can form a complex with glycoproteins and be actively released into the bloodstream to act as a signaling molecule in different signal transduction pathways [23], [24].

Numerous groups have demonstrated that the genetic and epigenetic alterations of cfDNA in cancer patients can be detected [25], and a possible role in genometastasis has been suggested [26], too. If the issues concerning the great variations in sensitivity and specificity and the mismatch between the cancer profiles from cfDNA studies and other methods [20], [27] were resolved then cfDNA monitoring could be a promising tool in cancer diagnostics.

### Foreign Sources of cfDNA

There is evidence that beyond the human cells of the subject other organisms can contribute to the cfDNA budget.

*Other humans:* Predominant donor origin was proved in patients receiving sex-mismatched bone marrow transplants using quantization of Y-chromosome sequences of plasma and serum cfDNA [28]. Cell free DNA of the fetus can be detected in maternal plasma promising non-invasive prenatal testing of fetal genetic conditions [29]. Though the fetal DNA is in relatively low concentration compared to the maternal cfDNA, fetal DNA has a lower molecular weight. With fragment size separation fetal DNA can be enriched [30] to a level that makes possible the diagnostics. Note, that in our study we use a similar technique and find that indeed different sized cfDNA fractions may have different origin.

#### Viruses

Virus DNA has been identified using plasma samples from different virus related (lung, gastric, head and neck cancer) tumor patients [31]–[33], however, the virus DNA concentration could not be related to the size of the solid tumor and no viral DNA could be identified in cervix cancer [34].

#### Bacteria

Using 16S rDNA analysis Jiang et. al [35] has shown, that the bacterial DNA level in the human plasma correlates with immune activation and the magnitude of immune restoration in antiretroviral-treated HIV infected persons. *Citrobacter freundii* and *Pseudomonas aeruginosa* sequences were identified from patients with acute pancreatitis by PCR and sequencing based approach [36].

#### Food

DNA from consumed food is usually not considered as a possible source of cfDNA since during food digestion all macromolecules are thought to be degraded to elementary constituents such as amino acids and nucleotides, which are then transferred to the circulatory system through several complex active processes [3]. Though, there are animal studies, mainly focusing on the GMO issue [4], supporting the idea that small fragments of nucleic acids may pass to the bloodstream and even get into various tissues. For example foreign DNA fragments were detected by PCR based techniques in the digestive tract and leukocytes of rainbow trouts fed by genetically modified soybean [37], and other studies report similar results in goats [38], pigs [39], [40] and mice [5].

## Results and Discussion

As a first step we have surveyed the composition of cfDNA in samples from 200 human individuals pooled into four groups based on colonoscopy diagnosis as having inflammatory bowel disease (IBD), adenoma (AD), colorectal cancer (CRC) or as negative (NEG). To avoid contamination we have used a contained blood collection and plasma separation system. During the nucleic acid isolation Laminar flow with HEPA filter and filtered pipetting tips were used. Since at the early stage we have separated DNA from particulates, the only possibility of contamination would have been in the form of free DNA which we find very improbable.

Since the sequencing technique produces relatively short fragments (50 nt) it is not possible to estimate the original fragment size from a sequencing study. To be able to infer the foreign cfDNA fragment distribution, prior to sequencing each sample has been separated into three fractions according to their average DNA length. Fraction 1 contained intact DNA above 10 kb (10 thousand base pairs), fraction 2 fragments between 200 bp to 10 kb (smear) and fraction 3 around 200 bp long segments (nucleosomal DNA). After barcoding, fragment libraries were sequenced on a SOLiD IV Next Generation Sequencing (NGS) system yielding 50 nt long reads a total of 86.6 Gbases. Sequencing data is publicly available here: http://www.ebi.ac.uk/ena/data/view/ERP002472. Despite the relatively short lengths of the NGS reads, the separation to fractions and barcoding made possible to identify the original size of the DNA fragments in the blood. On average 71.1% of the reads could be mapped to the human reference genome. The goal of the original study was to find (human) genetic differences between the four groups, according to the stage of their disease, but the relatively large amount of unmapped reads urged us to explore their origin, which is the subject of this article. With discarding the cellular DNA during the sample preparation, using cfDNA only and in this second step discarding the human-matching short reads we have achieved a significant enhancement on the detection of the possibly present non-human DNA.

Before searching for traces of foreign genomes we have discarded the reads which matched the reference human genome. In this way we have excluded most of the possible homologous sequence reads which could give false positive signal from low-complexity, repetitive or evolutionally conservative human sequences. During the initial alignment to human genome we have used permissive parameter settings (“-n 3”) of the Bowtie NGS aligner tool [41] that allowed alignments with several mismatches. This made possible to identify reads which had mutations compared to the reference genome or which had read errors during the sequencing process. On the other hand, during the alignment to foreign genomes, to reduce the possibility of chance alignments, we have used a more stringent criterion, the “-n 0” switch of Bowtie and accepted alignments only with perfect match in the first 28 nt long seed region. To further reduce the possibility of false positives and chance matches to homologous, evolutionally conservative human segments we have fitted the reads matching the tomato genome against the whole refseq genomic collection of NCBI using BLASTN [42] with default settings (*blast2 -p blastn -i sample.fa -d refseq_genomic -m 7 -o results.xml*). The resulting GenBank IDs were joined to the NCBI taxonomy database to associate them with classes and divisions.

**Figure 1:**
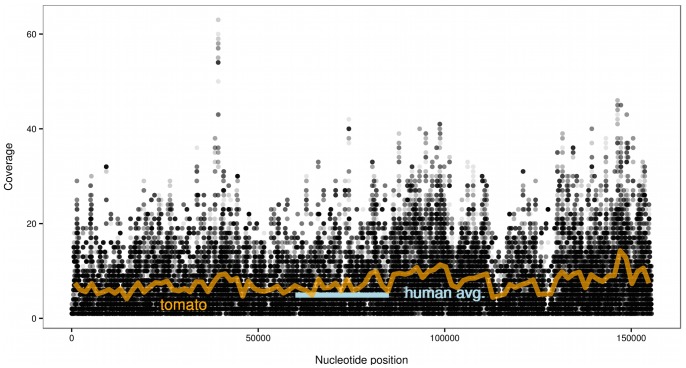
Coverage of the tomato chloroplast in the IBD sample. Small gray dots indicate the counts of alignments at individual nucleotide positions, darker shades are the result of several overlapping points. The orange line is the smoothed coverage of the tomato chloroplast, while the short gray dash indicates the average coverage level of the human genome for the same sample.

Testing the sequences against the chloroplast genome collection of NCBI (Table 1), over 25,000 sequence reads (Table 2) aligned to plant chloroplasts, among which *Solanum tuberosum* (potato) and/or the closely related *Solanum lycopersicum* (tomato) were the most abundant. Calculating the statistics for the tomato chloroplast alone, 127,885 of the 155,461 nucleotides in the plastome are covered by at least one read for the IBD sample. The average coverage is 6.3, which is higher than the sample's coverage of 4.9 for the human genome (see Figure 1). We have found hints for presence of DNA from other food related species (e.g. chicken), but due to the larger genetic homology between vertebrates, larger samples would be needed for convincing results, results will be discussed elsewhere.

**Table 1:** The accession numbers and names of the plants.

Accession number	Name	Accession number	Name
NC_008101.1	*Scenedesmus obliquus*	NC_008097.1	*Chara vulgaris*
NC_008099.1	*Oltmannsiellopsis viridis*	NC_008100.1	*Helicosporidium*
NC_008117.1	*Zygnema circumcarinatum*	NC_008116.1	*Staurastrum punctulatum*
NC_008114.1	*Pseudendoclonium akinetum*	NC_008115.1	*Eucalyptus globulus*
NC_008155.1	*Oryza sativa Indica Group*	NC_008235.1	*Populus alba*
NC_008289.1	*Ostreococcus tauri*	NC_008326.1	*Liriodendron tulipifera*
NC_008325.1	*Daucus carota*	NC_008334.1	*Citrus sinensis*
NC_008335.1	*Platanus occidentalis*	NC_008336.1	*Nandina domestica*
NC_001840.1	*Cyanidium caldarium*	NC_000925.1	*Porphyra purpurea*
NC_002186.1	*Mesostigma viride*	NC_001319.1	*Marchantia polymorpha*
NC_001320.1	*Oryza sativa Japonica Group*	NC_001568.1	*Epifagus virginiana*
NC_001675.1	*Cyanophora paradoxa cyanelle*	NC_001713.1	*Odontella sinensis*
NC_000926.1	*Guillardia theta*	NC_000927.1	*Nephroselmis olivacea*
NC_008359.1	*Morus indica*	NC_001799.1	*Toxoplasma gondii*
NC_002202.1	*Spinacia oleracea*	NC_008372.1	*Stigeoclonium helveticum*
NC_008407.1	*Jasminum nudiflorum*	NC_008408.1	*Bigelowiella natans*
NC_008454.1	*Pelargonium x hortorum*	NC_008456.1	*Drimys granadensis*
NC_008457.1	*Piper cenocladum*	NC_008535.1	*Coffea arabica*
NC_008588.1	*Phaeodactylum tricornutum*	NC_008589.1	*Thalassiosira pseudonana*
NC_008591.1	*Agrostis stolonifera*	NC_008590.1	*Hordeum vulgare*
NC_008602.1	*Sorghum bicolor*	NC_008641.1	*Gossypium barbadense*
NC_001666.2	*Zea mays*	NC_008788.1	*Nuphar advena*
NC_008796.1	*Ranunculus macranthus*	NC_008822.1	*Chlorokybus atmophyticus*
NC_008829.1	*Angiopteris evecta*	NC_002652.1	*Euglena longa*
NC_009143.1	*Populus trichocarpa*	NC_002694.1	*Lotus japonicus*
NC_009270.1	*Capsella bursa-pastoris*	NC_009259.1	*Phaseolus vulgaris*
NC_009269.1	*Barbarea verna*	NC_009273.1	*Lepidium virginicum*
NC_009275.1	*Nasturtium officinale*	NC_009266.1	*Aethionema grandiflorum*
NC_009267.1	*Olimarabidopsis pumila*	NC_009268.1	*Arabis hirsuta*
NC_009271.1	*Crucihimalaya wallichii*	NC_009272.1	*Draba nemorosa*
NC_009274.1	*Lobularia maritima*	NC_009265.1	*Aethionema cordifolium*
NC_002762.1	*Triticum aestivum*	NC_004677.2	*Pinus koraiensis*
NC_009573.1	*Rhodomonas salina*	NC_007898.2	*Solanum lycopersicum*
NC_009598.1	*Chloranthus spicatus*	NC_009601.1	*Dioscorea elephantipes*
NC_009600.1	*Illicium oligandrum*	NC_009599.1	*Buxus microphylla*
NC_009618.1	*Cycas taitungensis*	NC_009681.1	*Leptosira terrestris*
NC_009766.1	*Cuscuta reflexa*	NC_009765.1	*Cuscuta gronovii*
NC_009808.1	*Ipomoea purpurea*	NC_009949.1	*Cuscuta obtusiflora*
NC_009950.1	*Lolium perenne*	NC_009962.1	*Ceratophyllum demersum*
NC_009963.1	*Cuscuta exaltata*	NC_010093.1	*Acorus americanus*
NC_010109.1	*Lemna minor*	NC_002693.2	*Oenothera elata*
NC_010323.1	*Carica papaya*	NC_010358.1	*Oenothera argillicola*
NC_010359.1	*Aneura mirabilis*	NC_010360.1	*Oenothera glazioviana*
NC_010361.1	*Oenothera biennis*	NC_010362.1	*Oenothera parviflora*
NC_010433.1	*Manihot esculenta*	NC_010442.1	*Trachelium caeruleum*
NC_010548.1	*Cryptomeria japonica*	NC_003119.6	*Medicago truncatula*
NC_010601.1	*Guizotia abyssinica*	NC_010654.1	*Welwitschia mirabilis*
NC_003386.1	*Psilotum nudum*	NC_010772.1	*Heterosigma akashiwo*
NC_010776.1	*Fagopyrum esculentum*	NC_011032.1	*Brachypodium distachyon*
NC_011031.1	*Oedogonium cardiacum*	NC_011163.1	*Cicer arietinum*
NC_011395.1	*Babesia bovis T2Bo apicoplast*	NC_011600.1	*Vaucheria litorea*
NC_011828.1	*Trifolium subterraneum*	NC_011930.1	*Keteleeria davidiana*
NC_011942.1	*Gnetum parvifolium*	NC_011954.1	*Ephedra equisetina*
NC_012052.1	*Syntrichia ruralis*	NC_012099.1	*Pyramimonas parkeae*
NC_012097.1	*Pycnococcus provasolii*	NC_012101.1	*Monomastix sp. OKE-1*
NC_012224.1	*Jatropha curcas*	NC_012568.1	*Micromonas pusilla*
NC_012575.1	*Micromonas sp. RCC299*	NC_004115.1	*Chaetosphaeridium globosum*
NC_012615.1	*Megaleranthis saniculifolia*	NC_011157.3	*Pinus longaeva*
NC_011152.3	*Picea sitchensis*	NC_012898.1	*Aureococcus anophagefferens*
NC_012903.1	*Aureoumbra lagunensis*	NC_012818.1	*Alsophila spinulosa*
NC_012927.1	*Bambusa oldhamii*	NC_012978.1	*Parachlorella kessleri*
NC_011713.2	*Festuca arundinacea*	NC_013086.1	*Selaginella moellendorffii*
NC_013088.1	*Dendrocalamus latiflorus*	NC_013273.1	*Coix lacryma-jobi*
NC_013359.1	*Bryopsis hypnoides*	NC_013498.1	*Ectocarpus siliculosus*
NC_013553.1	*Parthenium argentatum*	NC_004543.1	*Anthoceros formosae*
NC_004561.1	*Atropa belladonna*	NC_013703.1	*Cryptomonas paramecium*
NC_013707.1	*Olea europaea*	NC_013823.1	*Typha latifolia*
NC_013843.1	*Vigna radiata*	NC_008096.2	*Solanum tuberosum*
NC_014062.1	*Anomochloa marantoidea*	NC_014063.1	*Lathyrus sativus*
NC_014057.1	*Pisum sativum*	NC_014056.1	*Oncidium Gower Ramsey*
NC_014267.1	*Kryptoperidinium foliaceum*	NC_014287.1	*Durinskia baltica*
NC_013991.2	*Phoenix dactylifera*	NC_014348.1	*Pteridium aquilinum*
NC_014340.1	*Chromera velia*	NC_014345.1	*Alveolata sp. CCMP3155*
NC_004766.1	*Adiantum capillus-veneris*	NC_001603.2	*Euglena gracilis*
NC_014346.1	*Floydiella terrestris*	NC_004799.1	*Cyanidioschyzon merolae*
NC_014592.1	*Cheilanthes lindheimeri*	NC_014569.1	*Erodium texanum*
NC_014573.1	*Geranium palmatum*	NC_014582.1	*Monsonia speciosa*
NC_014575.1	*Cedrus deodara*	NC_014589.1	*Cathaya argyrophylla*
NC_014570.1	*Eucalyptus grandis*	NC_014697.1	*Prunus persica*
NC_014699.1	*Equisetum arvense*	NC_014674.1	*Castanea mollissima*
NC_014675.1	*Isoetes flaccida*	NC_014676.1	*Theobroma cacao*
NC_004823.1	*Eimeria tenella*	NC_014808.1	*Thalassiosira oceanica*
NC_014807.1	*Corynocarpus laevigata*	NC_014874.1	*Rhizanthella gardneri*
NC_015083.1	*Erodium carvifolium*	NC_015104.1	*Smilax china*
NC_015113.1	*Anthriscus cerefolium*	NC_015084.1	*Coccomyxa sp. C-169*
NC_015139.1	*Brassica rapa s. pekinensis*	NC_004993.1	*Calycanthus floridus*
NC_011153.4	*Pinus contorta*	NC_011154.4	*Pinus gerardiana*
NC_011155.4	*Pinus krempfii*	NC_011156.4	*Pinus lambertiana*
NC_011158.4	*Pinus monophylla*	NC_011159.4	*Pinus nelsonii*
NC_015206.1	*Fragaria vesca*	NC_015204.1	*Gossypium thurberi*
NC_015308.1	*Hevea brasiliensis*	NC_015402.1	*Ptilidium pulcherrimum*
NC_015401.1	*Olea europaea*	NC_015403.1	*Fistulifera*
NC_015359.1	*Chlorella variabilis*	NC_015605.1	*Nelumbo lutea*
NC_015610.1	*Nelumbo nucifera*	NC_015604.1	*Olea europaea*
NC_015608.1	*Olea woodiana*	NC_015621.1	*Ageratina adenophora*
NC_015623.1	*Olea europaea subsp. maroccana*	NC_015543.1	*Jacobaea vulgaris*
NC_005086.1	*Amborella trichopoda*	NC_005087.1	*Physcomitrella patens*
NC_005353.1	*Chlamydomonas reinhardtii*	NC_005878.2	*Saccharum hybrid*
NC_005973.1	*Oryza nivara*	NC_006050.1	*Nymphaea alba*
NC_006084.1	*Saccharum officinarum*	NC_006137.1	*Gracilaria tenuistipitata*
NC_006290.1	*Panax ginseng*	NC_006861.1	*Huperzia lucidula*
NC_007144.1	*Cucumis sativus*	NC_007288.1	*Emiliania huxleyi*
NC_001631.1	*Pinus thunbergii*	NC_001865.1	*Chlorella vulgaris*
NC_000932.1	*Arabidopsis thaliana*	NC_007407.1	*Acorus calamus*
NC_007500.1	*Nicotiana sylvestris*	NC_007499.1	*Phalaenopsis aphrodite*
NC_007578.1	*Lactuca sativa*	NC_001879.2	*Nicotiana tabacum*
NC_007602.1	*Nicotiana tomentosiformis*	NC_007758.1	*Theileria parva*
NC_007932.1	*Porphyra yezoensis*	NC_007944.1	*Gossypium hirsutum*
NC_007943.1	*Solanum bulbocastanum*	NC_007942.1	*Glycine max*
NC_007957.1	*Vitis vinifera*	NC_007977.1	*Helianthus annuus*

The chloroplast genomes of these species were obtained from the NCBI archive and their presence was tested in the sequenced human samples.

**Table 2:** The initial number of sequence reads and the ones matching the chloroplast genome collection.

Sample ID	Fragmentsize (bp)	Total numberof reads	Bowtie*Chloroplast*	BLAST		
				Bacteria	Mammalia	Plantsonly
AD1	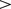 10 k	88,223,059	58 (0.657 ppm)	11	0	44
AD2	200…10 k	69,413,572	33 (0.475 ppm)	4	0	19
AD3	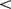 200	129,543,409	28 (0.216 ppm)	4	0	18
CRC1	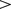 10 k	211,165,918	248 (1.174 ppm)	70	1	154
CRC2	200…10 k	214,141,527	261 (1.218 ppm)	42	0	193
CRC3	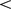 200	104,843,894	184 (1.754 ppm)	44	0	112
IBD1	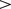 10 k	163,948,523	23319 (142.234 ppm)	820	39	21565
IBD2	200…10 k	148,952,652	237 (1.591 ppm)	32	2	190
IBD3	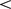 200	165,613,909	93 (0.561 ppm)	4	0	85
NEG1	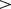 10 k	153,790,123	257 (1.67 ppm)	82	4	161
NEG2	200…10 k	137,374,304	275 (2.00 ppm)	47	1	215
NEG3	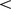 200	143,953,730	90 (0.625 ppm)	21	0	69

BLAST against the complete NCBI reference sequence database confirms that the chloroplast matching reads identified by the NGS alignment software Bowtie match only plant genomes and just in very few cases mammal genomes. The bacterial alignments are partly the result of the chloroplasts' genetic homology to them or may indicate the presence of circulating bacterial DNA. Among the IBD fractions, the one with largest DNA fragment size has the largest concentration.

The number of aligning short reads shows large differences between the various samples (see Table 2). Most of the matches are in the 1st fraction of IBD that contains the longest (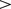
10 kb) intact DNA segments. This is surprising in the light of the current paradigm [43], which assumes that during digestion and absorption DNA is degraded to nucleotides. Our results show that not just some of the DNA can avoid the complete degradation, but fragments large enough to carry complete genes can pass from the digestive tract to blood. As shown in Table 2 the BLAST verification is consistent with the original findings, for chloroplast target sequences dominant part of BLAST hits matched plants only (i.e. not any other species in NCBI Ref. Seq.). The bacterial matching reads can be the result of the genetic homology of the chloroplast genome and bacterial genomes, or may indicate the presence of bacterial DNA in the samples.

All these results strengthen our conclusion that the meal-derived DNA fragments are able to avoid the total degradation in the gastrointestinal tract and enter the circulation through a previously unknown mechanism.

### Validation on Independent Samples

The NGS technology is evolving so fast and sequences are produced in such a rate that detailed understanding of all the information hiding in them cannot keep pace with data collection; hence already analyzed data may provide new insights for another research question. So, to confirm our discovery we have searched the publicly available NGS archives [44]–[46] for circulating cell-free DNA sequencing data. Compared to nuclear genome sequencing studies, plasma DNA data is very rare in the archives. We have found altogether 909 samples from 907 individuals in three studies with accession numbers DRP000446, SRP009039 and SRP016573. The analysis of these independent NGS data confirms our hypothesis that the presence of foreign DNA in human plasma is not unusual, though it shows large variation from subject to subject. SRP016573 study also provides a natural ‘negative control sample’ and eliminates the possibility that the results are mere statistical artifacts, since no trace of plant DNA was found in cord blood samples while more than 1000 reads were detected in the maternal plasma.

#### Independent sample from subject with inflammation shows high plant DNA concentration

**Figure 2:**
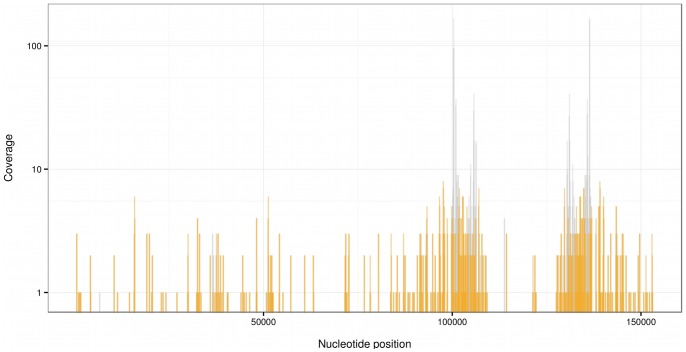
Brassica rapa chloroplast coverage pileup for the DRP000446 study. The gray spikes shows the counts of alignments at individual nucleotide positions (vertical scale is logarithmic). 27742 nucleotide positions of the total 180852 are covered. There are two regions around 100,000 and 135,000 where the coverage is more than 10 times than at other parts of the chloroplast. These are the regions where the ribosomal RNA genes are found which share very similar sequence with other chloroplasts and bacterial genomes. Indeed if we BLAST all the 1634 reads that matched the chloroplast genomes against the NCBI reference sequence database, 733 of them also match various bacterial genomes, but 897 does not match any other organisms, just plants. Removing those alignments that match bacterial genomes too, (gray spikes) makes the distribution more uniform.

The original goal of the DRP000446 study [47] was to detect potential pathogens in patients with Kawasaki disease, which is an autoimmune disease that involves the inflammation of blood vessels. The authors of the study have collected 6 DNA samples, two of them from formalin-fixed paraffin-embedded sample of the lymph node biopsy, one from pharyngeal swab sample and three form serum specimens at different stages of the disease. We have analyzed the sequencing data for the three serum samples DRR001355, DRR001356 and DRR001357. The total number of reads in the three samples is only 3.2 M which is much less than the 1732 M in our study, but since a different sequencer (Illumina Genome Analyzer II) was used, the reads are longer (81 nt long) than the 50 nt long reads in our studies (ABI Solid 4 System) reducing further the probability of false positives. Using the same pipeline as above, we have discarded the reads which match the human genome, then aligned the remaining ones to the chloroplast database. The largest number of unique positions were found for Brassica rapa (NC_015139) followed closely by orange (NC_008334). We provide the coverage map in Figure 2. 27742 nucleotide positions of the total 180852 are covered for Brassica rapa. Counting the multiply covered regions, the average coverage is 0.56. Note however, that the coverage is less uniform than for our IBD sample, the rRNA16 s and rRNA 23 s regions are overrepresented. This indicates that some of the matching DNA fragments may originate from some other related species which is missing from the chloroplast genome collection we use. Also, since the chloroplasts have been evolved from endosymbiotic bacteria, bacterial genome fragments may align to this evolutionarily conservative region. Indeed if we BLAST all the 1634 reads that matched the chloroplast genomes against the refseq database, 733 of them also match various bacterial genomes, but 894 does not match any other organisms, just plants. The coverage for Brassica rapa (orange spikes in Figure 2) without the bacterial reads is more uniform. Though in this sample the presence of the chloroplast genome is less definitive here than in our samples, the total reads vs. chloroplast matching reads ratio is even higher. The initial number of reads for the pooled IBD samples was 478 M and after BLAST filtering non-plant sequences there were 23649 matches for the chloroplast genomes i.e. 49 matches/million read (49 ppm), for the other 3 samples these ratios are around or below 1 ppm. For the DRP000446 sample the corresponding ratio is 1634/3.2 M = 497 ppm (272 ppm without bacterial tags). Note, that both in the IBD patients and the Kawasaki disease subject inflammation is present, hence from these samples we cannot exclude the possibility, that the presence of food DNA in high concentration is linked to inflammation.

#### The amount of plant DNA in 903 individual maternal plasma samples is log-normally distributed and hints diet pattern

Since the IBD sample was a pooled sample of 50 individuals we do not know how many individual samples contributed to the chloroplast matching reads. In the SRP009039 study [48] plasma DNA of 903 healthy pregnant women with ages ranging from 20 to 45 years were sequenced to study the possibility of prenatal noninvasive diagnosis of fetal trisomy. Depending on the platform, Illumina GAIIx and Illumina HiSeq 2000, the length of the reads are 36 nt or 50 nt, respectively. Though the individual read count is relatively small, typically in the 1 M–14 M reads/study range, the samples are individually identified, so compared to our pooled samples we hope to see if there are individual differences. As for the previous samples we have tested the presence of plant chloroplast DNA and got the largest coverage for soybean (Glycine max, NC_007942.1) with uniform coverage.

**Figure 3:**
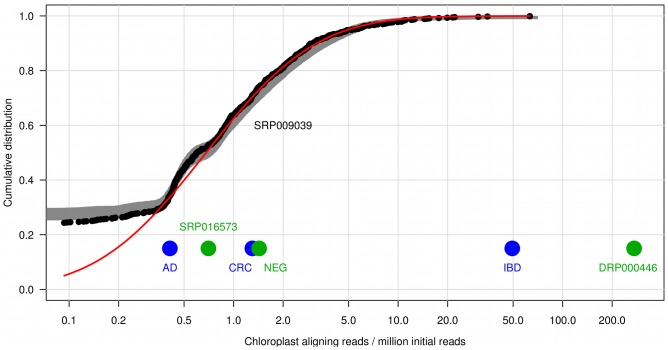
The cumulative distribution of plant DNA amount for over 900 subjects. It (black dots) can be fitted with log-normal distribution (red curve) above the sensitivity cutoff (0.35). The gray shaded band is the result of the simulation of 300 realizations of the log-normal process with taking into account the varying sizes of the samples. Among the independent samples (larger dots), the ones from patients with inflammatory diseases (IBD, DRP000446) have the largest concentration. For the SRP016573 sample only the maternal plasma concentration is shown, full blood samples with 0.001 ppm and 0.004 ppm and cord blood samples with zero alignments are omitted from the figure.

The overall average chloroplast DNA ratio is 1.481 ppm but there is a very large variation from sample to sample, so we visualize their cumulative distribution on a logarithmic scale in Figure 3. The numbers of reads per sample are in the range of 940,929–12,827,703 with an average 2,483,480, so it is not possible to detect concentration below 0.078 ppm for the largest, and below 0.35 ppm for the average sized sample. In 75% of the samples we could detect plant DNA and for 220 of the total 903 subjects there are no aligning reads at all, most probably because of the wide distribution and the low coverage. Down to the above mentioned cutoff value the data can be fitted with the following log-normal distribution:
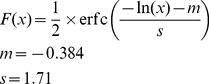
(1)with only two free parameters, the location parameter 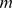 and the scale parameter 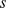, the analogs of mean and standard deviation, respectively. If we take into account the finite size of the samples even the cutoff break around 0.35 can be modeled. The gray shaded band in Figure 3 is the result of the simulation of 300 realizations of the log-normal process with taking into account the concrete sizes of the samples. Though log-normal distribution is ubiquitous in almost all disciplines [49] the precise agreement between the data and model is quite surprising. The trend may be explained by the exponential decay dynamics of foreign cfDNA with randomly varying half-lives or waiting times between consumption and blood sample collection.

**Figure 4:**
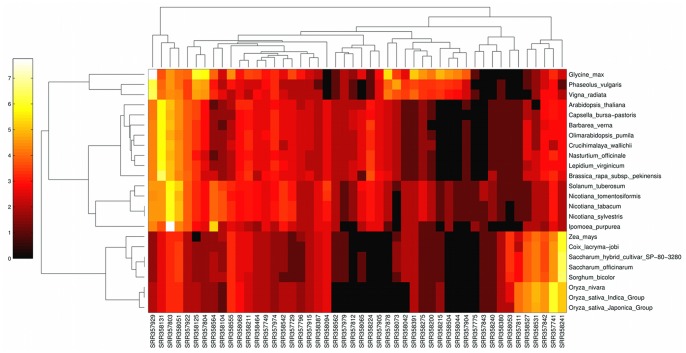
This heatmap shows the number of chloroplast matching reads on a 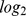 scale for the SRP009039 study. From the total 903 subjects the ones with the largest number of matches are shown (only the plant genomes with more than 50, and only the subjects with more than 10 matching reads), the rows are the plant species, the columns are the samples. The automatic clustering recovers the related plant species and the subjects can be also grouped by the food types.

There are alignments to several plant species and since the samples are from over 900 different subjects we can test the individual differences. This can be considered as a test of contamination too. If the food origin of this external DNA is true, we expect different plants dominating different samples, according to the different diet of patients, while lab contamination would most probably result the same composition in all samples. In Figure 4 we show how the number of matching reads are distributed between subjects and different plant species. To make the visualization of the broad distribution possible, only plants with at least 50 and samples with at least 10 aligning reads are shown. The clustering algorithm recovers the taxonomic groups of plants. The first three species (beans) are members of the Fabaceae family, the next eight species belong to the Brassicaceae family. These two families are distantly related in the Eurosids clade. There are four members from the Solanaceae family (potato, tobacco,) and one from the Convolvulaceae (Ipomoea, Cuscuta) family. These two families are members of the Solanales order. The remaining eight species are from the Poaceae family from the Monocots clade [50]. All these 24 plants are often consumed by humans or are close relatives of frequently eaten species while many non-edible plants which were part of the aligned chloroplast database do not show up on the list (see Table 1 for complete list of aligned species). Note that on one hand not edible but genetically related species can show up, and the other hand not all the frequently eaten plant species are part of our chloroplast genome collection. We suspect that the only outlier, the non-edible *Ipomoea purpurea* (morning glory) shows up because the similarity to the genome of *Ipomoea batatas* (sweet potato) or *Ipomoea aquatica* (kangkong, or Chinese spinach), a common ingredient in Southeast Asian dishes. Though the number of reads is too small to reconstruct the diet of the individuals, subjects with high Poaceae, high Fabaceae and “high everything except Poaceae” levels can be grouped together. We consider this pattern in food genome coverage as a further proof that the signal is not a statistical artifact.

#### Cord blood is free of plant DNA while it can be detected in mother's plasma

Four samples from fetal umbilical cord blood, maternal plasma and mother's and father's peripheral blood, with 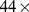
sequence depth were analyzed in the SRP016573 study to noninvasively infer fetal genotype and haplotype and identify Mendelian-disorder genes and complex disease-associated markers [51]. The setup of this study is ideal for testing plant DNA. According to our previous results we expect to find some plant DNA in the maternal plasma sample. Though peripheral full blood samples should contain traces of plant DNA, we expect it's relative concentration much smaller due to the higher amount of human DNA from blood cells. Though the maternal blood reaches the fetal chorion, and fetal plasma can be detected in mother's blood there is no direct fluid exchange between mother and fetus. So, even though some plasma DNA may have passed from mother to child, it's concentration would be much smaller in cord blood. We can use the cord blood sample as a natural ‘negative control’: if the plant DNA signal was the result of some contamination during the processing or a statistical artifact, it should show up in this sample, too. Beyond high sequencing depth, the paired layout of this study with 2
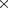
100 nt long reads (Illumina HiSeq 2000) further diminish the chance of false positives.

While 1110 reads (0.703 ppm) from the maternal plasma aligned to chloroplast genomes, only 3 reads (0.004) from the father's and 1 read (0.001 ppm) from the mother's full blood sample matched them. There was not a single chloroplast matching read among the 560 M from the umbilical cord blood (see Table 3).

**Table 3:** The number of sequence reads in the samples and the number and ratio of chloroplast matching ones.

Study accession number	Total number of reads	Bowtie *Chloroplast*	BLAST		
			Bacteria	Mammalia	Plants only
AD	287,180,040	119 (0.414 ppm)	19	0	81
CRC	530,151,339	693 (1.307 ppm)	156	1	459
IBD	478,515,084	23649 (49.421 ppm)	856	41	21840
NEG	435,118,157	622 (1.429 ppm)	150	5	445
DRP000446	3,284,956	1634 (497.419 ppm)	733	5	894
SRP009039	2,551,402,380	3781 (1.481 ppm)	908	20	2746
SRP016573 maternal plasma	1,578,181,738	1110 (0.703 ppm)	169	12	851
SRP016573 father full blood	672,693,854	3 (0.004 ppm)	0	0	2
SRP016573 mother full blood	696,977,900	1 (0.001 ppm)	0	0	1
SRP016573 cord blood	560,035,524	0 (0.000 ppm)	0	0	0

Though some reads which were identified by the aligner software (Bowtie) can be fitted to other species like bacteria, most of them match plants only.

### Conclusion

The analysis of **all the publicly available** circulating cell-free DNA sequencing data of over 1000 human subjects confirms our hypothesis that the presence of foreign DNA in human plasma is not unusual. It shows large variation from subject to subject following strikingly well a log-normal distribution with the highest concentration in patients with inflammation (Kawasaki disease, IBD). These findings could lead to a revision of our view of degradation and absorption mechanisms of nucleic acids in the human body.

## Materials and Methods

Here we describe the details for the NEG, IBD, AD, CRC samples. For DRP000446, SRP009039 and SRP016573 samples see the cited papers and the description at the archives, respectively.

Anticoagulated blood was collected from the antecubital veins of fifty healthy individuals (median age, 42.6 years) who had negative colonoscopy. Blood was also collected from three diseased groups including inflammatory bowel disease (48) (median age, 35.2 years) colorectal adenoma (35) (median age, 53.4 years) and colorectal cancer (37) (median age, 64.9 years) with positive macroscopic and pathological finding. Study plan of the medical research was made according to the current legislations and World Medical Association Declaration of Helsinki. Ethics Committee approval was obtained (Nr.: TUKEB 2009/037. Semmelweis University Regional and Institutional Committee of Science and Research Ethics, Budapest, Hungary) and written informed consent was provided by all patients.

### Plasma Separation and Cell Free DNA Isolation

Whole-blood samples were collected into Vacutainer tubes (BD Medical Systems) and plasma separation was performed by double centrifugation method (2×1500 g for 10 min) at 4°C within 1 h of the blood collection. The purified plasma fraction was stored at −80°C. From plasma cfDNA was extracted using QIAamp Circulating Nucleic Acid Kit (Qiagen) following the manufacturer's instructions with modification. Briefly, cfDNA was isolated from 5 ml plasma without addition carrier RNA. Quantification of cfDNA was performed using Qubit dsDNA HS Assay fluorometric Kit (Invitrogen). Eluates were pooled from equivalent amount from each sample and concentrated to a final volume of 200 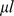 using QIAamp Circulating Nucleic Acid Kit (Qiagen). To achieve the optimal sample volume (50 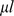) SpeedVac (Eppendorf) concentrator was used.

### SOLiD Fragment Library Preparation and Sequencing

The DNA fragment library resequencing was performed on SOLiD IV system. Total of 3–5 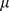
g cfDNA was pooled from each group and three fractions were separated via electrophoresis using SyberSafe 1% TBE agarose gel (Invitrogen) and recovered by QIAquick Gel Extraction Kit (Qiagen). Three fractions were separeted according to sequence lengths: the 1st fraction is the intact DNA above 10 kb, the second is between 200 bp to 10 kb (smear) and the 3rd fraction is around 200 bp (nucleosomal DNA). In case of fractions 1 and 2 physical fragmentation was optimized and performed by Covaris S2 instrument. The fractions were labeled by individual barcode (Life Technologies). The size selected DNA (100 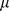
L) was end-repaired by adding 40 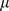
L 5× End-Polishing Buffer, 4 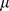
L dNTP mix, (10 mM), 2 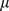
L End Polishing Enzyme 1, 10 U
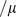
L, 8 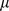
L End Polishing Enzyme 2, 5 U
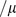
L, 46 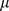
L MQ distilled water in 200 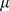
l total volume and incubating for 30 min at room temperature. The DNA (180 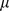
L) was purified by AMPure XP beads (70 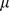
L) (Agencourt). Nick translation and amplification (15 cycles) was performed to amplify the ligated and purified DNA using Platinum PCR Amplification Mix (Life Technologies). Distribution of the amplified and non-ligated DNA was controlled using Agilent High Sensitivity DNA kit (Agilent). Size selection was performed using E-gel 2% system (Invitrogen) with the following parameters: required size: 200–250 bp, iBase program: Run E Gel DC, run time: 16 min, 200 ng 50 bp ladder. Library quantification was performed by TaqMan assay and ePCR followed. After the quantification 7×10^8^ beads were loaded into the sequencing slides. The sequencing yielded 50 nt long reads – a total of 86.6 Gbases.
